# Comparative analysis of revision causes between robotic-assisted and conventional manual unicompartmental knee arthroplasty: a systematic review and meta-analysis

**DOI:** 10.1186/s43019-026-00311-x

**Published:** 2026-02-26

**Authors:** Pengyu Xiang, Hongfu Jin, Yiming Dai, Yixin Yang, Chenhao Jia, Lather Sagar, Jian Zhang, Yungang Wu, Chunwu Zhang, Shengwu Yang, Zhenhan Deng

**Affiliations:** 1 Department of Orthopedics, The First Affiliated Hospital of Wenzhou Medical University, Wenzhou, 325000 Zhejiang China; 2 Geriatrics Center, The First Affiliated Hospital of Wenzhou Medical University, Wenzhou, 325000 Zhejiang China; 3 Sports Medicine Center, West China Hospital, Sichuan University, Chengdu, 610041 Sichuan China; 4 Department of Epidemiology, University of California, Los Angeles, CA 951772 USA; 5 Department of Spine Surgery, Shenzhen Second People’s Hospital, The First Affifiliated Hospital of Shenzhen University, Shenzhen, 518035 Guangdong China; 6 Department of the Orthopedics of TCM, The First Affiliated Hospital of Wenzhou Medical University, Wenzhou, 325000 Zhejiang China

**Keywords:** Unicompartmental knee arthroplasty, Revision, Robot, Meta-analysis, Systematic review

## Abstract

**Background:**

Robotic-assisted unicompartmental knee arthroplasty (R-UKA) is an emerging procedure; however, its benefits over conventional manual unicompartmental knee arthroplasty (C-UKA) are controversial, especially the revision and failure rates, and existing studies failed to reach a consensus on this issue.

**Methods:**

The literature search was conducted on four databases (PubMed, Embase, Cochrane Library and Web of Science) from inception to 28 April 2025 according to the guidelines for Preferred Reporting Items for Systematic Reviews and Meta-Analysis (PRISMA). Eligibility criteria were studies that were written in English and reported any causes for a revision or failure subsequent to UKA with comparisons between R-UKA and C-UKA. The quality of each article was assessed using the Cochrane collaboration risk of bias tool or the Newcastle–Ottawa Scale.

**Results:**

A total of 15 studies incorporating 29,982 patients with 30,099 knees (22,290 in the C-UKA group and 7809 in the R-UKA group) were analyzed. Compared with R-UKA, C-UKA showed higher total revision rates (RR: 1.58; 95% CI: ~1.33–1.87; *P* < 0.00001; *I*^2^ = 43%). Prosthesis loosening, infection, pain, and progression of disease were the main reasons for R-UKA revision, whereas for C-UKA revision, loosening, progression of disease, infection, and limb malalignment were the major causes. Loosening was the predominant reason in both groups across all follow-up periods; early revisions were also due to infection and disease progression. Within 2–5 years, the secondary reasons differed, being limb malalignment for C-UKA and pain for R-UKA.

**Conclusions:**

Compared with C-UKA, R-UKA may lower the risk of revision related to loosening, disease progression, and limb malalignment. Loosening remains the primary revision cause for both. Large-scale prospective trials with unified technical details are warranted to draw more rigorous conclusions in the future.

**Trial registration:**

PROSPERO CRD420251042604.

**Supplementary Information:**

The online version contains supplementary material available at 10.1186/s43019-026-00311-x.

## Background

Unicompartmental knee arthroplasty (UKA) is an effective treatment option for unicompartment knee osteoarthritis (OA), and with the rapid technological evolution in orthopedic surgery, robotic-assisted UKA (R-UKA) has gradually drawn attention. By optimizing bone resection, implant positioning, and the accuracy of alignment with the assistance of robotic devices and computer technology both pre- and intra-operatively [1, 38], R-UKA not only provides a new surgical method for clinical practice but also reduces cutting errors, rendering orthopedists with more precise manipulation than conventional manual UKA (C-UKA) [2, 3]. Despite the advantages of R-UKA, there are several latent drawbacks of this procedure such as substantial costs, additional radioactive scanning, extra training for manipulation and the long learning curve, in addition to the prolonged operative time and the extra incisions for the registration pins potentially increase the risk of infection [1, 4]. Thus, the clinical benefits of R-UKA over conventional techniques remain empirically contested, especially the revision and failure rates, and existing studies failed to draw definitive conclusions on this issue. An review by MacNeille [39] concluded that Batallier et al. [5] compared the annual revision rate for both groups of patients, finding that R-UKA had a lower revision rate than C-UKA, whereas Wong et al. [6] demonstrated that R-UKA had vague advantages over the conventional technique and was associated with higher revision rates; therefore, the technical potential of R-UKA remains to be validated.

To the best of our knowledge, there has been no study that has systematically analyzed the difference between the two types of UKA with regard to the cause of revision. Hence, the primary objective of this study is to systematically review and synthesize the existing comparative evidence on revision causes between R-UKA and C-UKA to determine whether there exist discrepancies in the revision rate and major causes of revision between the two types of UKA. On the basis of the results of current studies, we hypothesize that, compared with R-UKA, C-UKA may be associated with a higher overall revision rate and specifically with an increased risk of revision due to malalignment or loosening.

## Method

### Protocol registration

This study was performed in accordance with the guidelines and standards set by the Preferred Reporting Items for Systematic Reviews and Meta-Analyses (PRISMA) [7], and the protocol of the study was registered at the International Prospective Register of Systematic Reviews (ID: CRD420251042604).

### Selection criteria

Eligibility criteria were delineated as follows: (1) studies that reported any causes for a revision or failure subsequent to UKA presented in terms of number of cases in each group. A revision was defined as the exchange, removal, or addition of any component of the prosthesis, a second UKA operation or conversion to total knee arthroplasty (TKA); (2) comparisons were between R-UKA and C-UKA, and the term “R-UKA” comprised both active robotic platforms and computer navigation systems. (3) The full-text content and outcome data of the included articles were required to be accessible and complete.

Studies were excluded if they met any of the following criteria: (1) non-English language; (2) simultaneous combination of UKA with other knee surgeries (e.g., meniscectomy, ligament reconstruction, osteotomy); (3) animal or cadaveric experiments; (4) article types including case report, review, conference abstract, meta-analysis, letter, editorial, or comment; or (5) data from the same patients that were reported in another study with longer follow-ups.

### Data sources and search strategy

All of the relevant studies were searched in the databases, including PubMed, Embase, Cochrane Library and Web of Science, from inception to 28 April 2025, with no restriction to time span. The English search terms included “robot,” “unicompartmental,” “unicondylar,” “partial,” “knee,” “arthroplasty,” and “replacement,” which were combined using the Boolean operators AND or OR. The detailed search strategies tailored for each database were presented in Supplementary File 1.

### Selection process

After removing duplicate records, two independent reviewers screened titles and abstracts of the remaining records to exclude unnecessary articles preliminarily, as well as the full-text review for those records reckoned as relevant. Disagreements were resolved and discussed by a third independent reviewer until reaching consensus on approval among the three reviewers.

### Data extraction

The data were extracted by two independent researchers using a standard data extraction form, and a third person intervened to mediate any disagreement. The main contents of data extraction included first author, year of publication, journal, level of evidence, number of patients, number of knees, number of revision knees, age, gender, duration of follow-up, reported reasons for revision, the type of robot, and any other relevant information.

### Assessment of risk of bias and quality of evidence

The Cochrane collaboration risk of bias tool [8] was used to assess the quality of randomized controlled trials (RCTs). Two researchers independently completed this process according to seven items: (1) random sequence generation, (2) allocation concealment, (3) blinding of participants and personnel, (4) blinding of outcome assessment, (5) incomplete outcome, (6) selective reporting, and (7) other bias. Each item was classified as “low risk,” “unclear risk,” or “high risk of bias.” Review Manager (version 5.4.1) was applied to assess and visualize the quality and bias of RCTs. With regard to cohort studies and case–control studies, the risk of bias and methodological quality was evaluated by using the Newcastle–Ottawa Scale (NOS) [9], which incorporates three domains: (1) selection, (2) comparability, (3) outcome (for cohort studies) or exposure (for case–control studies). Each domain had at least one item, and each study could acquire a maximum of nine points, with scores levels classified as follows: 7–9 (high quality), 4–6 (mediate quality), 0–3 (low quality). The level of evidence of the included studies was assessed according to the criteria from the Oxford Centre for Evidence-Based Medicine.

### Data synthesis and analysis

Statistical analyses were performed using Review Manager (version 5.4.1) and STATA (version 17.0). Dichotomous variables such as the incidence of revision were evaluated using the Mantel–Haenszel (MH) method, and the pooled effect sizes were depicted with risk ratio (RR) value and their 95% confidence intervals (CIs). Heterogeneity of the included studies was quantified using *I*^2^index [10]. According to the Cochrane’s handbook, a random-effect model would be employed if the value of *I*^2^was > 50%, which meant a significant heterogeneity was observed. Otherwise, a fixed-effect model would be utilized for meta-analysis (*I*^2^ ≤ 50%). Subgroup analyses were performed on the basis of different follow-up periods, types of study, types of robot, and different reasons for revision. We considered a *P*-value less than 0.05 to be statistically significant. The funnel plot was introduced to visualize the heterogeneity between studies, which was further verified by utilizing Begg’s test and Egger’s test [11] (no significant publication bias was detected; suppose the *P* values for Begg’s test and Egger’s test were > 0.05).

## Result

### Literature selection

**Fig. 1:**
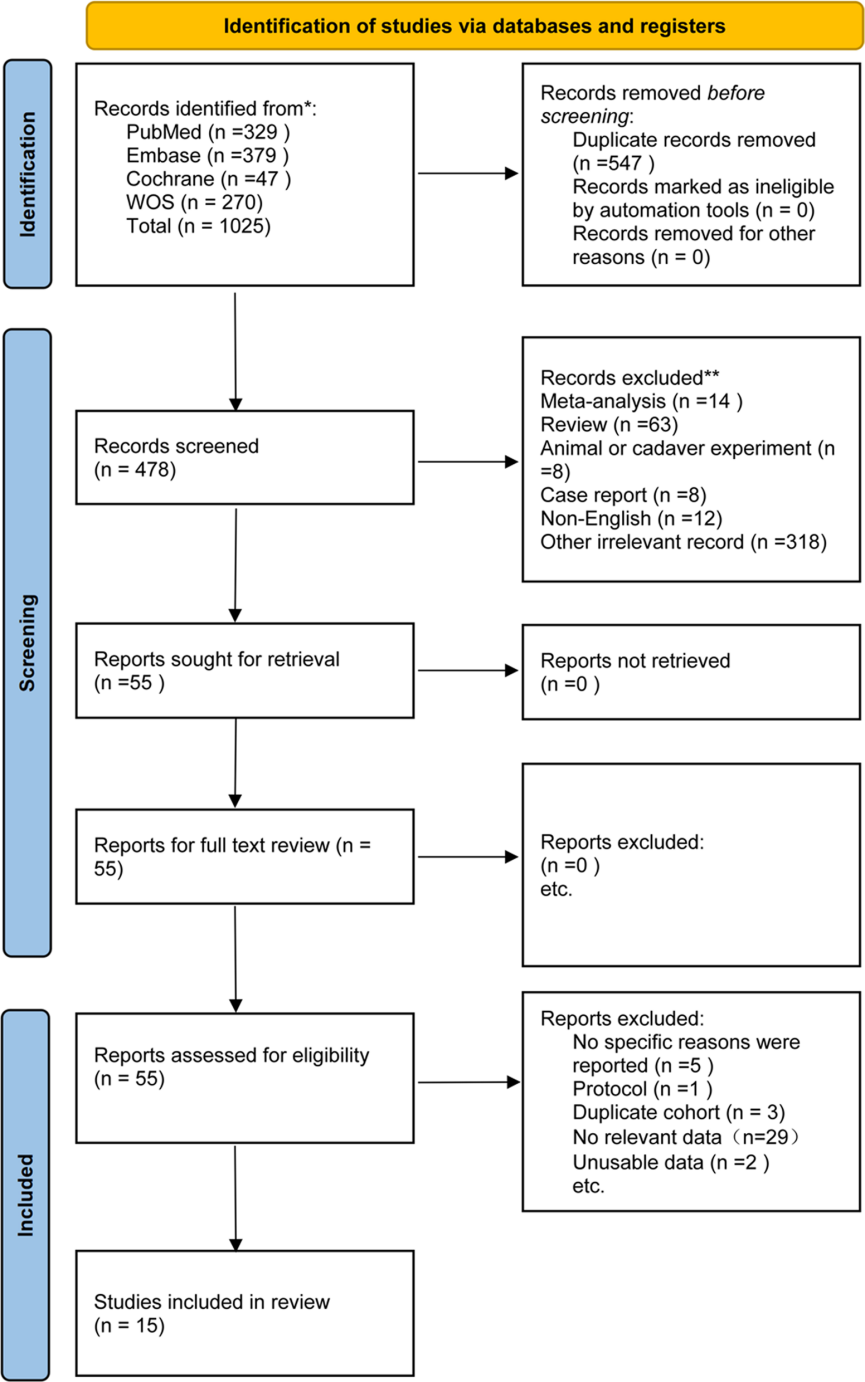
PRISMA flow diagram of literature screening

The PRISMA flow diagram of the meta-analysis is depicted in Fig. 1. Initial database searches yielded 1025 records; after systematic and manual removal of 547 duplicated records, 478 articles were screened by title and abstract for further retrieval. Following retrieval, 55 full-text articles were retrieved and assessed for eligibility. Ultimately, 15 articles were included in the systematic review and meta-analysis based on predefined inclusion and exclusion criteria.

### Basic characteristics of the literature

The general characteristics of the included studies are depicted in Table 1 (pages 22–25). A total of 15 articles (9 were cohort studies, 2 were RCTs, and 4 were case–control studies) were eventually included in the statistical analysis, incorporating 29,982 patients with 30,099 knees (22,290 in the C-UKA group and 7809 in the R-UKA group). St. Mart et al. [12] presented the most comprehensive reasons for revisions with a sample size larger than 10,000 knees (9561 for C-UKA and 2851 for R-UKA), while Guild et al. [13] provided the largest sample among the included studies with a total of 15,742 knees (11,766 for C-UKA and 3976 for R-UKA). The mean follow-up period for the participants ranged from 6.4 months to 18 years, and the mean age ranged from 60.7 to 71.2 years. The publication year of the included studies ranged from 2014 to 2025, 73.3% of which were published since 2020. MAKO and BlueBelt Navio were the two most frequently used robotic systems, while only one study [14] described BrainLAB navigation as a system of assistance. In terms of the level of evidence of the 15 included studies, 12 were level III, 1 was level IV and 2 were level I.

**Table 1:** Characteristics of the included articles

Study	Journal	Type of study	No. of patients (M/F)	Follow-up period, months	Mean age	No. of knees (*n*)	No. of medial UKA cases (*n*)	No. of lateral UKA cases (*n*)	No. of revisions (*n*)	Type of robot	Reported reasons for revision	Level of evidence
Batailler 2018 [5]	*Knee Surg Sports Traumatol Arthrosc*	Case–control	151 (NR)	C-UKA: 24.2 ± 16 R-UKA:19.7 ± 9	C-UKA: 68 ± 10 R-UKA:69 ± 9.6	160 C-UKA: 80 R-UKA: 80	114 C-UKA: 57 R-UKA: 57	46 C-UKA: 23 R-UKA: 23	11 C-UKA: 7 R-UKA: 4	Navio	Aseptic loosening with implant malposition, aseptic loosening without implant malposition, limb malalignment, unexplained pain	**III**
Blyth 2025 [15]	*Bone Joint J*	RCT	129 (71/58)	120	C-UKA: 62.5 (43–92) R-UKA: 62.1 (43–92)	129 C-UKA: 65 R-UKA: 64	129 C-UKA: 65 R-UKA: 64	0 C-UKA: 0 R-UKA: 0	5 C-UKA: 5 R-UKA: 0	Mako	Tibial loosening, progression of lateral osteoarthritis, pain	**I**
Cécile Batailler 2023 [16]	*Knee Surg Sports Traumatol Arthrosc*	RCT	66 (33/33)	C-UKA: 6.4 ± 0.7 R-UKA: 6.5 ± 0.7	C-UKA: 67.1 ± 8.1 R-UKA: 65.6 ± 7.9	66 C-UKA: 33 R-UKA: 33	66 C-UKA: 33 R-UKA: 33	0 C-UKA: 0 R-UKA: 0	1 C-UKA: 0 R-UKA: 1	Navio	Unexplained pain	**I**
Foissey 2023 [17]	*Int Orthop*	Retrospective cohort	356 (150/206)	61.3 ± 24.0 C-UKA: 76.1 ± 30.3 R-UKA: 49.4 ± 13.4	67.4 ± 7.9 C-UKA: 68.3 ± 8.1 R-UKA: 66.7 ± 7.7	356 C-UKA: 159 R-UKA: 197	356 C-UKA: 159 R-UKA: 197	0 C-UKA: 0 R-UKA: 0	26 C-UKA: 20 R-UKA: 6	Navio	Tibial aseptic loosening, femoral loosening, lateral OA, malalignment, tibial overhang, tibial plateau fracture	**III**
Hansen et al. 2014 [24]	J *Arthroplasty*	Retrospective cohort	62 (27/35)	> 24	C-UKA: 60.66 ± 11.78 R-UKA: 57.13 ± 9.81	62 C-UKA: 32 R-UKA: 30	62 C-UKA: 32 R-UKA: 30	0 C-UKA: 0 R-UKA: 0	1 C-UKA: 1 R-UKA: 0	Mako	Deep postoperative infection	**III**
G. Guild et al. 2025 [13]	*Arthroplast Today*	Retrospective cohort	15,742 (7434/8308)	24	C-UKA: 63.0 ± 9.1 R-UKA: 63.0 ± 9.2	15,742 C-UKA: 11,766 R-UKA: 3976	NR C-UKA: NR R-UKA: NR	NR C-UKA: NR R-UKA: NR	382 C-UKA: 304 R-UKA: 78	NR	Aseptic revision, periprosthetic joint infection	**III**
Maritan 2023 [18]	*Knee Surg Sports Traumatol Arthrosc*	Retrospective cohort	95 (17/78)	C-UKA: 90.3 ± 9.1 R-UKA: 95.4 ± 11.0	C-UKA: 61.5 ± 8.5 R-UKA: 60.9 ± 8.4	95 C-UKA: 43 R-UKA: 52	0 C-UKA: 0 R-UKA: 0	95 C-UKA: 43 R-UKA: 52	5 C-UKA: 3 R-UKA: 2	Mako	Aseptic loosening, patellofemoral OA, periprosthetic fracture, inexplicable pain	III
Guillaume 2020 [19]	*Knee Surg Sports Traumatol Arthrosc*	Case–control	354 (NR)	C-UKA: 30.2 ± 23.4 R-UKA: 22.5 ± 12.9	C-UKA: 67.1 ± 10.7 R-UKA: 66.7 ± 9.3	391 C-UKA: 191 R-UKA: 200	294 C-UKA: 135 R-UKA: 159	97 C-UKA: 56 R-UKA: 41	29 C-UKA: 21 R-UKA: 8	Navio	Aseptic loosening without implant malposition, aseptic loosening with implant malposition, contralateral/patellofemoral OA, limb malalignment, subsidence, infection	III
Wong 2019 [6]	*Knee Surg Sports Traumatol Arthrosc*	Retrospective cohort	176 (74/102)	C-UKA: 45.6 ± 22.8 R-UKA: 33.6 ± 10.8	C-UKA: 67.9 ± 9.5 R-UKA: 70.4 ± 9.7	176 C-UKA: 118 R-UKA: 58	176 C-UKA: 118 R-UKA: 58	0 C-UKA: 0 R-UKA: 0	15 C-UKA: 8 R-UKA: 7	Mako	Consistent pain, aseptic loosening, instability, periprosthetic joint infection, periprosthetic tibia fracture	III
Andriollo 2024 [14]	*Knee*	Retrospective cohort	52 (13/39)	216.4 C-UKA: 221.8 ± 11.9 R-UKA: 210.8 ± 5.9	C-UKA: 66.8 ± 5.6 R-UKA: 65.8 ± 6.8	52 C-UKA: 26 R-UKA: 26	52 C-UKA: 26 R-UKA: 26	0 C-UKA: 0 R-UKA: 0	3 C-UKA: 1 R-UKA: 2	BrainLAB Navigation	Aseptic loosening, progression of arthritis, polyethylene wear,	III
Lau et al. 2024 [20]	*Arthroplasty*	Case–control	140(38/102)	36	C-UKA: 70.9 ± 7.9 R-UKA: 69.6 ± 7.3	140 C-UKA: 82 R-UKA: 58	140 C-UKA: 82 R-UKA: 58	0 C-UKA: 0 R-UKA: 0	3 C-UKA: 1 R-UKA: 2	Navio	Aseptic loosening, unexplained pain	III
Canetti 2018 [21]	*Arch Orthop Trauma Surg*	Retrospective cohort	41 (NR)	NR	NR	44 C-UKA: 24 R-UKA: 20	0 C-UKA: 0 R-UKA: 0	44 C-UKA: 24 R-UKA: 20	3 C-UKA: 3 R-UKA: 0	Navio	Aseptic loosening, impingement	III
St Mart 2020 [12]	*Bone Joint J*	Retrospective cohort	12,412 (7091/5321)	C-UKA: 22.68 ± 13.44 R-UKA: 16.80 ± 10.68	C-UKA: 65.2 ± 9.6 R-UKA: 65.7 ± 9.3	12,412 C-UKA: 9561 R-UKA: 2851	NR C-UKA: NR R-UKA: NR	NR C-UKA: NR R-UKA: NR	348 C-UKA: 301 R-UKA: 47	Mako	Loosening, progression ofdisease, bearing dislocation, fracture, infection, pain, instability, malalignment, prosthesis dislocation, incorrect sizing, patellofemoral pain, implant breakage tibial, lysis, metal related pathology, osteonecrosis, synovitis, other	III
Rossi et al. 2025 [22]	*Arthroplast Today*	Retrospective cohort	63 (30/33)	21.9 ± 11.6 C-UKA: 22.25 ± 12.2 R-UKA: 21.7 ± 11.4	71.2 ± 6 C-UKA: 71.2 ± 5.4 R-UKA: 71.2 ± 6.1	126 C-UKA: 36 R-UKA: 90	126 C-UKA: 36 R-UKA: 90	0 C-UKA: 0 R-UKA: 0	3 C-UKA: 1 R-UKA: 2	Mako/Navio	Tibial loosening, contralateral osteoarthritis progression, anterior cruciate ligament mucoid degeneration, postoperative overcorrection,	IV
Yeung et al. 2023 [23]	*Arthroplasty*	Retrospective case–control	140(NR)	18.1 ± 6.1	NR	148 C-UKA: 74 R-UKA: 74	148 C-UKA: 74 R-UKA: 74	0 C-UKA: 0 R-UKA: 0	4 C-UKA: 1 R-UKA: 3	Mako	Tibial component loosening	III

NR, not reported

### Quality assessment and publication bias

**Fig. 2:**
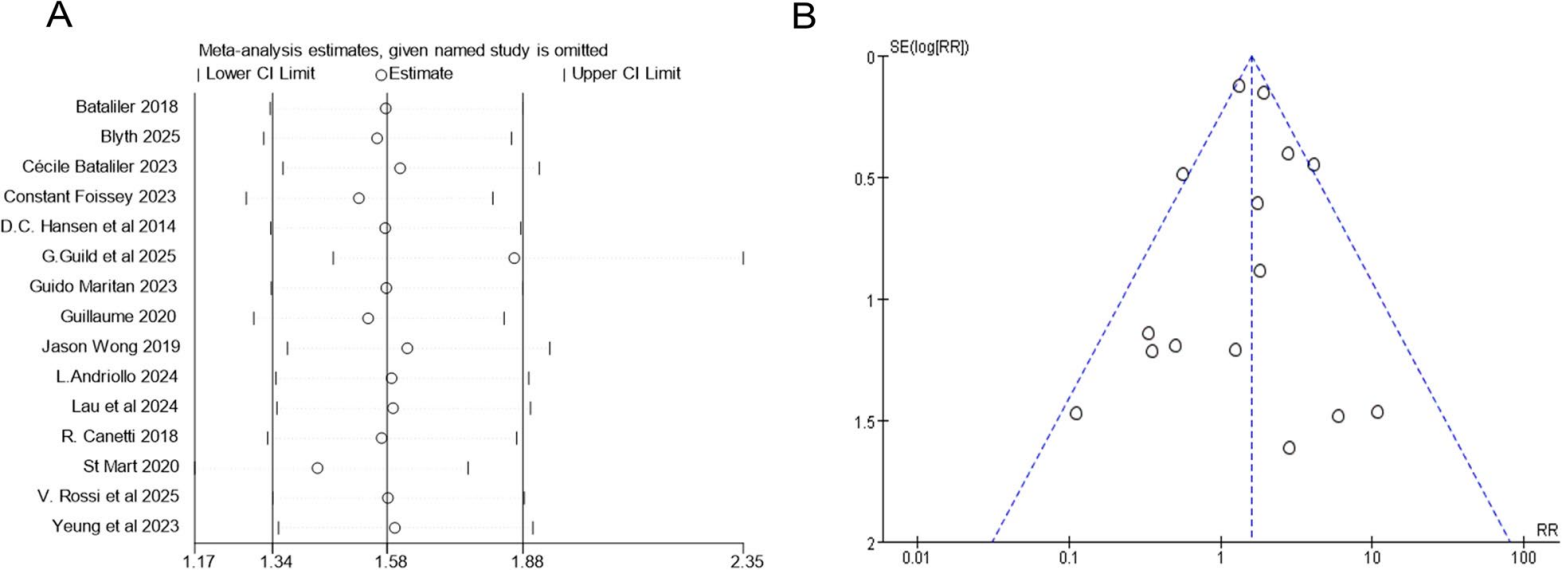
**A** Sensitivity analysis of the included studies. **B** Funnel plot of the included studies

For RCTs, the Cochrane collaborative bias tool was utilized, and the results are depicted in Supplementary File 2. Blyth’s study [15] was reckoned to have a low risk of bias, while Cecile’ study [16] showcased a high risk of bias in terms of the process of blinding. As for the cohort studies and case–control studies, the NOS scale scores were used, ranging from eight to nine among cohort studies and from six to eight among case–control studies; only one article received the lowest score of six (Supplementary File 2). A sensitivity analysis of the included studies was applied, in which one study was excluded and the rest were analyzed to assess whether the outcomes could be altered remarkably by a single study. The result indicates that the outcomes of synthesized analysis exhibit stability when excluding any specific study. Particularly, by excluding Guild [13], the result changed comparatively more notably than by omitting the others (Fig. 2A). A funnel plot of the included studies was also generated, with no significant asymmetry being observed, which suggests the absence of publication bias in the meta-analysis (Fig. 2B). Begg’s test and Egger’s test were implemented to further detect the presence of bias, and the *P*-values were 0.767 for Begg’s test and 0.799 for Egger’s test, both showing no presence of publication bias (Supplementary File 3).

### Total revision rates and revision reasons

**Fig. 3:**
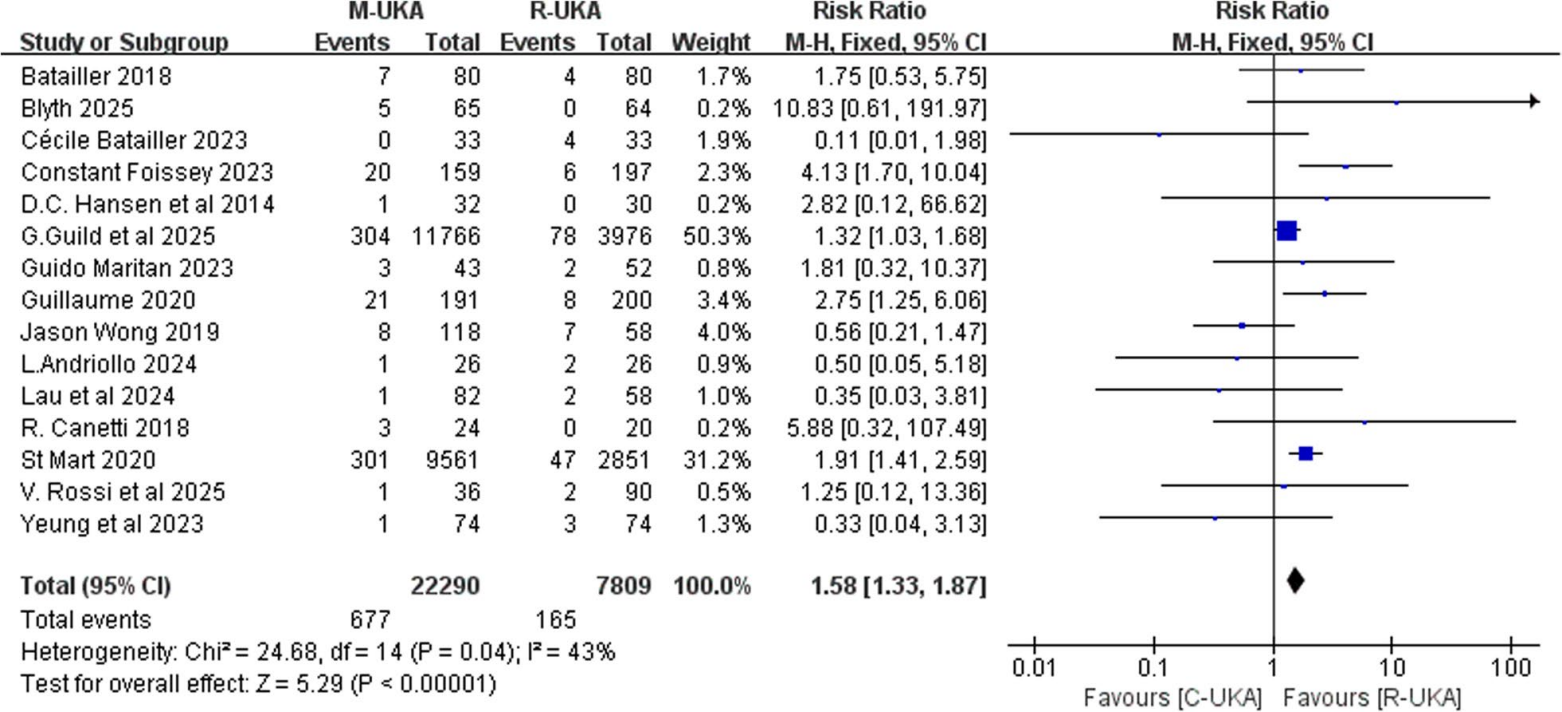
Forest plot of total revision

**Fig. 4:**
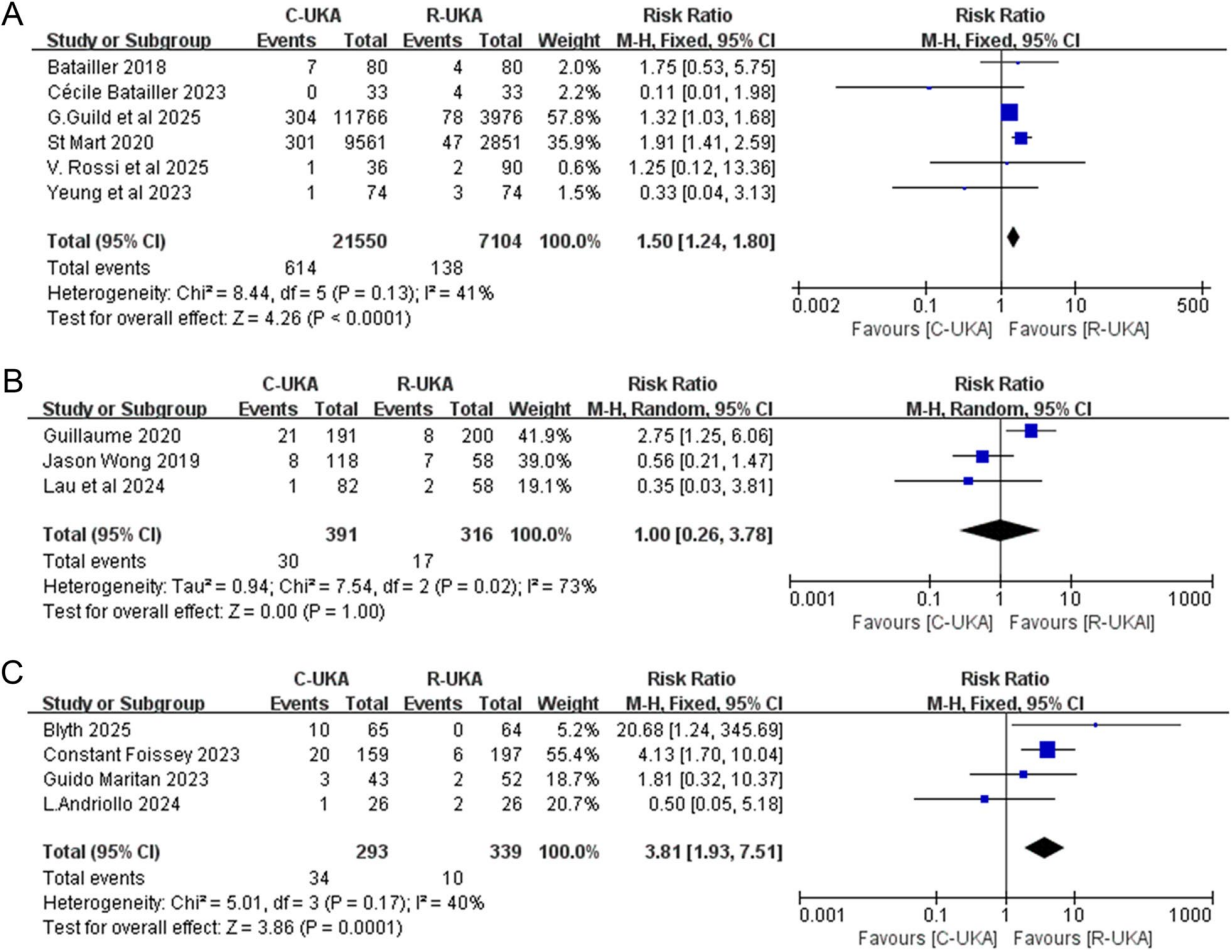
Forest plot of total revision stratified by follow-up period. **A** Follow-up period ≤ 2 years.** B** Follow-up between 2 and 5 years.** C** Follow-up period > 5 years

A total of 15 studies reported the number of knees and the number of revised knees with detailed reasons, comprising 22,290 knees in the C-UKA group and 7809 knees in the R-UKA group. The results of the meta-analysis demonstrated that C-UKA was associated with a significantly higher total revision rate compared with R-UKA (RR: 1.58; 95% CI: ~1.33–1.87; *P* < 0.00001; *I*^2^ = 43%, Fig. 3). Stratification by follow-up duration revealed that both the ≤ 2-year cohort (RR: 1.50; 95% CI: 1.24–1.80; *P* < 0.0001; *I*^2^ = 41%, Fig. 4A) and the > 5-year cohort (RR: 3.81; 95% CI: 1.93–7.51; *P* < 0.0001; *I*^2^ = 40%, Fig. 4C) exhibited significant differences between two groups and indicated a statistically higher total revision rate of C-UKA. Conversely, no statistically significant difference was observed in the 2–5-year follow-up stratum using the random-effects model (RR: 1.00; 95% CI: 0.26–3.78; P = 1.00; I^2^ = 73%, Fig. 4B). Subgroup analysis was also performed according to the type of study, which indicated that the major heterogeneity came from the two RCT studies (Supplementary File 4).

With regard to the concrete reasons for a revision, the most reported reason was loosening, which appeared in ten articles in the C-UKA group and eight articles in the R-UKA group. Moreover, progression of disease, pain, infection, and limb malalignment were also several major reasons for a revision, as shown in Supplementary File 5 along with the incidence within the first 5 years. The detailed number and reasons for revision of each study according to follow-up period are specified in Table 2. Within the first 2 years, loosening, infection and progression of disease were the predominant reasons in both groups. During the 2–5-years period, loosening remained the leading cause for both, with limb malalignment in the C-UKA and pain in the R-UKA serving as the second major reason, respectively. In the long-term (> 5 years), loosening persisted as the most common reason in both groups.

**Table 2:** Causes of revision in each study according to follow-up period

Follow-up period	Study	Reported reasons for revision	C-UKA	R-UKA
≤ 2 years				
	Batailler 2018	Aseptic loosening with implant malposition, aseptic loosening without implant malposition, limb malalignment, unexplained pain	Aseptic loosening with implant malposition 3 Limb malalignment 3 Unexplained pain 1	Aseptic loosening without implant malposition 3 Unexplained pain 1
	Cécile Batailler 2023	Unexplained pain	None	Pain 1
	Guild et al. 2025	Aseptic revision, periprosthetic joint infection	Aseptic revision 279 periprosthetic joint infection 25	Aseptic revision 73 periprosthetic joint infection 5
	St. Mart 2020	Loosening, progression of disease, bearing dislocation, fracture, infection, pain, instability, malalignment, prosthesis dislocation, incorrect sizing, patellofemoral pain, implant breakage tibial, lysis, metal related pathology, osteonecrosis, synovitis, other	Loosening 114 Progression of disease 64 Bearing dislocation 25 Fracture 26 Infection 25 Pain 18 Instability 8 Malalignment 8 Prosthesis dislocation 3 Incorrect sizing 3 Implant breakage 1 Lysis 1 Metal related pathology 1 Osteonecrosis 1 Synovitis Other 2	Loosening 10 Progression of disease 8 Fracture 1 Infection 18 Pain 4 Instability 3 Malalignment 1 Other 2
	Rossi et al. 2025	Tibial loosening, contralateral osteoarthritis progression, anterior cruciate ligament mucoid degeneration, postoperative overcorrection,	Postoperative overcorrection and contralateral osteoarthritis progression 1	Tibial loosening 1 Anterior cruciate ligament mucoid degeneration 1
	Yeung et al. 2023	Tibial component loosening	Tibial component loosening 1	Tibial component loosening 3
2–5 years				
	Guillaume 2020	Aseptic loosening without implant malposition, aseptic loosening with implant malposition, contralateral/patellofemoral OA, limb malalignment, subsidence, infection	Aseptic loosening without implant malposition 3 Aseptic loosening with implant malposition 6 Limb malalignment 10 Subsidence 1 Contralateral/patellofemoral OA 1	Aseptic loosening without implant malposition 3 Aseptic loosening with implant malposition 1 Subsidence 2 Infection 1 Contralateral/patellofemoral OA 1
	Wong 2019	Consistent pain, aseptic loosening, instability, periprosthetic joint infection, periprosthetic tibia fracture	Consistent pain 4 Aseptic loosening 1 Instability 1 Periprosthetic joint infection 1, periprosthetic tibia fracture 1	Consistent pain 3 Aseptic loosening 3 Instability 1
	Lau et al. 2024	Aseptic loosening, unexplained pain	Aseptic loosening 1	Unexplained pain 1 Aseptic loosening 1
> 5 years				
	Blyth 2025	Tibial loosening, progression of lateral osteoarthritis, pain	Pain 2 Tibial loosening 1 Progression of lateral osteoarthritis 2	None
	Foissey 2023	Tibial aseptic loosening, femoral loosening, lateral OA, malalignment, tibial overhang, tibial plateau fracture	Tibial aseptic loosening 13 Femoral loosening 2 Lateral OA 2 Malalignment 2 Tibial overhang 1	Tibial aseptic loosening 5 Tibial plateau fracture 1
	Maritan 2023	Aseptic loosening, patellofemoral OA, periprosthetic fracture, inexplicable pain	Aseptic loosening 2 Persistent symptoms 1	Patellofemoral OA 1 Inexplicable pain 1
	Andriollo 2024	Aseptic loosening, progression of arthritis, polyethylene wear (With no description in which group)	1	2
Other				
	Canetti 2018	Aseptic loosening, impingement	Aseptic loosening 2 Impingement 1	None
	Hansen et al. 2014	Deep postoperative infection	Deep postoperative infection 1	None

Other: studies with not reported follow-up or follow-up without specific grouping

#### Loosening

**Fig. 5:**
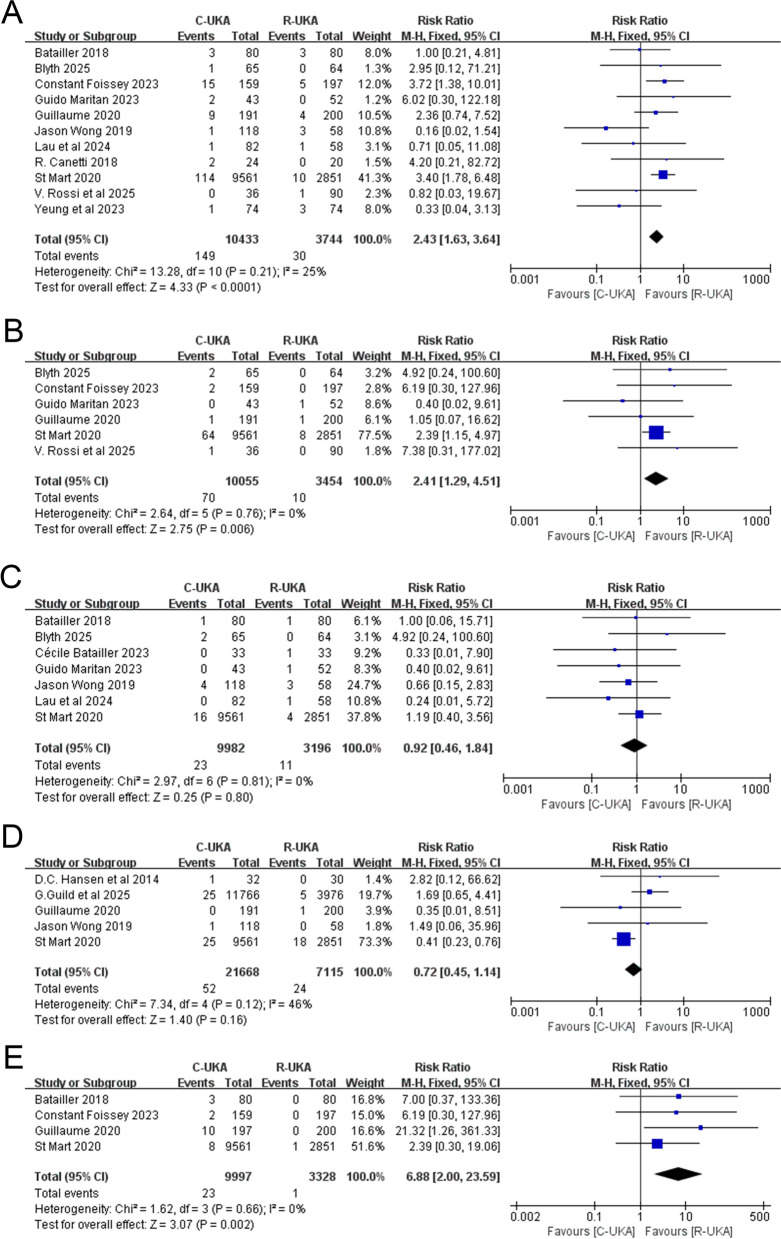
**A** Forest plot of revision caused by loosening. **B** Forest plot of revision caused by progression of disease. **C** Forest plot of revision caused by pain. **D** Forest plot of revision caused by infection. **E** Forest plot of revision caused by limb malalignment

In total, 11 studies [5, 6, 12, 15, 17–23] reported loosening as a cause of revision, including aseptic loosening, tibial loosening, and femoral loosening, with a total sample size of 10,433 knees in the C-UKA group and 3744 in the R-UKA group, respectively. The results of the meta-analysis showcased a significantly higher risk of revision caused by loosening for patients receiving C-UKA when compared with R-UKA (RR: 2.43; 95% CI: ~1.63–3.64; *P* < 0.0001; *I*^2^ = 25%, Fig. 5A).

#### Progression of disease

A total of six studies [12, 15, 17–19, 22] listed the progression of disease as a reason for revision, involving 10,055 knees in the C-UKA group and 3454 knees in the R-UKA group. The analysis revealed noteworthy disparities in the revision rates between C-UKA and R-UKA, suggesting that C-UKA has a higher likelihood of revision caused by progression of disease (RR: 2.41; 95% CI: ~1.29–4.51; *P* = 0.006; *I*^2^ = 0%, Fig. 5B). In our analysis, the progression of disease included progression of contralateral or patellofemoral OA.

#### Pain

A revision resulting from pain was discussed in seven studies [5, 6, 12, 15, 16, 18, 20], incorporating 9982 knees in the C-UKA group and 3196 knees in the R-UKA group, with no heterogeneity being found (*I*^2^ = 0%, *P* = 0.81). Furthermore, the results suggested no significant differences of revision rates between C-UKA and R-UKA (RR: 0.92; 95% CI: ~0.46–1.84; *P* = 0.80, Fig. 5C).

#### Infection

Data from 5 studies [6, 12, 13, 19, 24] involving 21,688 knees in the C-UKA group and 7115 knees in the R-UKA group presented the revision cases incurred by infection. The forest plot showed no significant differences of revision rates between the two groups (RR: 0.72; 95% CI: ~0.45–1.14; *P* = 0.16; *I*^2^ = 46%, Fig. 5D).

#### Limb malalignment

Revision due to limb malalignment was mentioned in four articles [5, 12, 17, 19], with 9997 knees in the C-UKA group and 3328 knees in the R-UKA group. The results revealed a significant difference, indicating that C-UKA had a much higher revision rate than R-UKA (RR: 6.88; 95% CI: ~2.00–23.59; *P* = 0.002; *I*^2^ = 0%, Fig. 5E).

### Revision rates of UKA with different types of bearing.

Among the 15 included studies, there were 4 studies [6, 14, 18, 20] involving 269 knees in the C-UKA group and 194 knees in the R-UKA group that only assessed fixed-bearing UKA between the conventional and the robotic technique. Regarding the total revision rate, the results indicated that fixed-bearing C-UKA was associated with lower risk of revision compared with fixed-bearing R-UKA, but no statistical difference was observed (RR: 0.67; 95% CI: ~0.32–1.39; *P* = 0.28; *I*^2^ = 0%). The rest of the 11 studies mixed fixed-bearing and mobile-bearing together for evaluation or gave no explicit descriptions of bearing types, and the results of total revision rates demonstrated that patients in the C-UKA group were associated with a significantly higher total revision rate compared with those in the R-UKA group (RR: 1.64; 95% CI: ~1.38–1.96; *P* < 0.00001; *I*^2^ = 43%) (Supplementary File 6).

## Discussion

This study successfully attained its objective, and the results support our primary hypothesis. The most prominent finding is that C-UKA was associated with a significantly higher risk of total revision compared with R-UKA, and the risk of revision was significantly greater in the C-UKA group for specific causes, including loosening, progression of disease and limb malalignment.

For R-UKA, loosening, infection, pain, and progression of disease are the several main reasons for revision, whereas for C-UKA, loosening, progression of disease, infection, and limb malalignment are the leading causes. By facilitating the precision of manipulation, R-UKA provides more accuracy of joint line alignment and implant positioning, which may contribute to a reduced likelihood of malalignment, prosthesis loosening, and subsequent revision surgery as evidenced by our results. Despite all this, loosening remains the most common reason for a revision surgery for both groups (0.7% for C-UKA and 0.4% for R-UKA, respectively); this finding is consistent with a study by Lachance et al. [25] evaluating patients undergoing conversion from UKA to TKA, among whom aseptic loosening accounted for the majority of the revision cohort.

Regarding revision attributed to progression of disease, namely the progression of contralateral or patellofemoral OA, the present study found a significant difference between C-UKA and R-UKA, which was in favor of the robot technique. One possible explanation for this finding is that R-UKA allows precise component positioning, force line alignment, and soft tissue balance, which are crucial for achieving optimized pressure distribution, averting excessive local cartilage wear in the contralateral compartment caused by the load imbalance [26, 27]. A distinction should be made between “limb malalignment” and “progression of disease” as revision causes. Malalignment is a direct technical failure, often identified as the primary reason for revision, whereas disease progression is a complex, long-term biological outcome influenced by multiple factors; suboptimal alignment serves as one such indirect cause, and even if not severe enough to directly cause a revision, it may elevate revision risk by altering joint biomechanics over time, thereby indirectly accelerating disease progression to a point requiring surgical intervention. Hernigou et al. [28] in their article assessing the impact of alignment on wear of the cartilage and implants pointed out that the overcorrection of valgus was associated with a higher risk of progression of OA in the opposite compartment, and the optimal angle of hip–knee–ankle was between 171° and 179° for medial implants. For UKA revised to TKA, the tibiofemoral component alignment was also a risk factor for wear that clinicians to which should attach emphasis [29].

Published systematic reviews and meta-analyses have also evaluated the revision rates of the two operations. A literature review in 2019 by Zhang et al. [30] analyzed a total of three studies that reported the revision rate, showcasing that R-UKA had relatively lower revision rates, but no significant difference was found between the two groups. In 2024, a meta-analysis with more trials and a larger sample size by Bensa et al. [2] yielded the same result, whereas the difference still did not reach statistical significance. In contrast, the present meta-analysis not only is in line with the previous but also is of statistical significance concerning total revision rates.

Although R-UKA is reported to improve surgical precision, most included studies with follow-up periods shorter than 5 years report aseptic loosening as the main cause of failure, which may be ascribed to several factors. For the procedure itself, the precision advantage of R-UKA is mainly reflected in the osteotomy and implant positioning stage, whereas the final fixation of the prosthesis still depends on the traditional bone cement technology, which is a crucial variable independent of robot technology and is reported to be associated with an increased rate of loosening compared with the cementless technique [31, 32]. Inappropriate cement thickness at the tibial bone–implant surface, uneven cement force distribution on the tibial component, and inadequate cement penetration into the trabecular bone may indirectly contribute to potential loosening, thereby offsetting the benefits of bone cutting accuracy [33–35]. Regarding patients, Lachance et al. deduced that the population with high body mass index (BMI) may be more susceptible to aseptic loosening [25], and Yeung et al. indicated that small knees were associated with increased risks of aseptic loosening owing to smaller surface area and components [23]. Moreover, patients undergoing UKA surgery are usually elderly and may be accompanied by poor bone quality such as osteoporosis, which is also a potential factor [36].

A notable aspect is that, among the incorporated studies in our meta-analysis, the majority reported that the surgeries were performed by experienced or senior surgeons, which implies that our findings may not be directly applicable to nonproficient surgeons since R-UKA requires a mean learning curve of 11 cases and longer operating time compared with C-UKA [4]. Moreover, during the initial learning phase of R-UKA, the nonproficiency and prolonged time of operation may increase the risk of infection, which may account for why C-UKA showed a relatively lower rate of revision triggered by infection in this study, though not reaching statistical difference [37]. Particularly, in our study, an article by Andriollo describes the BrainLAB VectorVision uni-knee navigation system as an assisting technique for UKA surgery, which is utilized in an imageless way and compared with the manual group; thus, this study was deemed as eligible. Nevertheless, to address the risk of bias, an attempt was made to exclude this study while conducting data analysis so as to ascertain whether it may significantly impact the stability of the pooled outcomes. Fortunately none of the outcomes were notably affected.

While by stratifying according to the bearing type, fixed-bearing C-UKA displayed relatively lower risk of revision compared with fixed-bearing R-UKA, no significant differences were observed, and only four studies were included, which should be cautiously interpreted. More studies are required to provide more information to supplement evidence. Wong et al., the author of the one of the four studies, inferred that the high revision rates of R-UKA in their study may be associated with the learning curve [6].

To the best of our knowledge, the present study is the first to conduct a systematic review and meta-analysis on revision causes between R-UKA and C-UKA, which provides an overview of the current evidence and offers valuable insight to surgeons. Nevertheless, it is essential to explicitly acknowledge certain limitations of our study. The most significant constraint is the limited number of high-quality prospective trials, particularly RCTs, which inevitably introduces greater risk of potential bias and confounding factors due to retrospective design and nonrandomization. Moreover, in our meta-analysis, UKA was categorized as a single surgical procedure for the purpose of comparison; however, actually, the UKA in the included studies encompassed assorted surgical instruments, techniques, robotic systems, bearing types, and prosthesis design, each may have distinct clinical outcomes and failure mechanisms, which was difficult to control for owing to the limited number of eligible literature and may introduce bias, thereby affecting the generalizability of the findings to specific UKA procedure. Finally, while most UKA procedures were performed in the medial compartment of the knee joint and the majority of the included studies exclusively evaluated the medial UKA cases, a few studies mixed medial UKA and lateral UKA together for report, which could not be separately evaluated, preventing a meaningful subgroup analysis and unavoidably introducing heterogeneity. Therefore, future studies with larger sample sizes, high quality and more unified technical details are warranted to rigorously investigate the specific technical nuances of UKA and their impact so as to provide more accurate comparative evidence.

## Conclusions

This study successfully achieved its objective, and the results provide strong support for our primary hypothesis, finding that compared with C-UKA, R-UKA may lower the risk of revision related to loosening, disease progression, and limb malalignment. Moreover, loosening remains the primary revision cause in both. Given the scarcity of high-quality evidence available and the distinctions of technique details, the conclusions are not definitive and should be discreetly interpreted. More large-scale prospective trials with unified technical details are warranted to draw more rigorous conclusions in the future.

## Supplementary Information

Additional file1 (DOCX 12 KB) Retrieval strategy.

Additional file2 (DOCX 149 KB) Results of methodological assessment.

Additional file3 (DOCX 87 KB) Results of Begg’s test and Egger’s test.

Additional file4 (DOCX 14 KB)Results of subgroup analysis

Additional file5 (DOCX 21 KB) Ranking of the causes.

Additional file6 (DOCX 13 KB) Revision rates of UKA with different types of bearing.

## Data Availability

All data generated or analyzed during this study are included in this published article and its supplementary materials.

## Abbreviations

CI: Confidence interval
C-UKA: Conventional manual unicompartmental knee arthroplasty
MH: Mantel–Haenszel
NOS: Newcastle–Ottawa Scale
OA: Osteoarthritis
RCT: Randomized controlled trial
RR: Risk ratio
R-UKA: Robotic-assisted unicompartmental knee arthroplasty
TKA: Total knee arthroplasty
UKA: Unicompartmental knee arthroplasty
