# Supplementary material for: Comparative analysis of revision causes between robotic-assisted and conventional manual unicompartmental knee arthroplasty: a systematic review and meta-analysis
Source: Knee Surg Relat Res. 2026 Feb 26;38:10. doi: 10.1186/s43019-026-00311-x (PMC12937531; doi:10.1186/s43019-026-00311-x)
Supplement: Supplementary file 1 — Additional file1 (DOCX 12 KB) Retrieval strategy. [file 43019_2026_311_MOESM1_ESM.docx]

**PubMed**

#1 (robot*[tiab])

#2 (Arthroplasty, Replacement, Partial Knee[tiab]) OR (Unicompartmental Knee Arthroplasty[tiab]) OR (Arthroplasty, Unicompartmental Knee[tiab]) OR (Knee Arthroplasty, Unicompartmental[tiab]) OR (Unicompartmental Knee Replacement[tiab]) OR (Knee Replacement, Unicompartmental[tiab]) OR (Partial Knee Replacement[tiab]) OR (Knee Replacement, Partial[tiab]) OR (Unicondylar Knee Replacement[tiab]) OR (Knee Replacement, Unicondylar[tiab]) OR (Unicondylar Knee Arthroplasty[tiab]) OR (Arthroplasty, Unicondylar Knee[tiab]) OR (Knee Arthroplasty, Unicondylar[tiab]) OR (Partial Knee Arthroplasty[tiab]) OR (Arthroplasty, Partial Knee[tiab]) OR (Knee Arthroplasty, Partial[tiab])

#3 #1 AND #2 329

**Web of science**

#1 TS=(robot*)

#2 (((((((((((((((TS=(Arthroplasty, Replacement, Partial Knee)) OR TS=(Unicompartmental Knee Arthroplasty)) OR TS=(Arthroplasty, Unicompartmental Knee)) OR TS=(Knee Arthroplasty, Unicompartmental)) OR TS=(Unicompartmental Knee Replacement)) OR TS=(Knee Replacement Unicompartmental)) OR TS=(Partial Knee Replacement)) OR TS=(Knee Replacement, Partial) OR TS=(Unicondylar Knee Replacement)) OR TS=(Knee Replacement, Unicondylar)) OR TS=(Unicondylar Knee Arthroplasty)) OR TS=(Arthroplasty, Unicondylar Knee)) OR TS=(Knee Arthroplasty, Unicondylar)) OR TS=(Partial Knee Arthroplasty)) OR TS=(Arthroplasty, Partial Knee)) OR TS=(Knee Arthroplasty, Partial)

#3 #1 AND #2 270

**Cochrane**

#1 (Arthroplasty, Replacement, Partial Knee):ti,ab,kw OR (Unicompartmental Knee Arthroplasty):ti,ab,kw OR (Arthroplasty, Unicompartmental Kknee):ti,ab,.kw OR(Knee Arthroplasty, Unicompartmental):ti,ab,kw OR (Unicompartmental Knee Replacement):ti,ab,kw 414

#2 (Knee Replacement, Unicompartmental):ti,ab,kw OR (Partial Knee Replacement):ti,ab,kw OR (Knee Replacement, Parial):ti,ab,kw OR (Unicondylar knee Replacement):ti,ab,kw OR (Knee Replacement, Unicondylar):ti,ab,kw 433

#3 (Unicondylar Knee Arthroplasty):ti,ab,kw OR (Arthroplasty, Unicondylar Knee):ti,ab,kw OR (Knee Arthroplasty, Unicondylar):ti,ab,kw OR (Partial Knee Arthroplasty):ti,ab,kw OR (Arthroplasty, Partial Knee):ti,ab,kw 311

#4 (Knee Arthroplasty, Partial):ti,ab,kw 268

#5 #1 OR #2 OR #3 OR #4 611

#6 robot* 8712

#7 #5 AND #6 47

**EMBASE**

#1 ‘arthroplasty, replacement, partial knee’ OR ‘unicompartmental knee arthroplasty’/exp OR ‘unicompartmental knee arthroplasty’ OR ‘arthroplasty, unicompartmental knee’ OR ‘knee arthroplasty, unicompartmental’ OR ‘unicompartmental knee replacement’/exp OR ‘unicompartmental knee replacement’ OR ‘knee replacement, unicompartmental’ OR ‘partial knee replacement’/exp OR ‘partial knee replacement’ OR ‘knee replacement, partial’ OR ‘unicondylar knee replacement’/exp OR ‘unicondylar knee replacement’ OR ‘knee replacement, unicondylar’ OR ‘unicondylar knee arthroplasty’/exp OR ‘unicondylar knee arthroplasty’ OR ‘arthroplasty, unicondylar knee’ OR ‘knee arthroplasty, unicondylar’ OR ‘partial knee arthroplasty’/exp OR ‘partial knee arthroplasty’ OR ‘arthroplasty, partial knee’ OR ‘knee arthroplasty, partial’ 3672

#2 ‘robot*’ 167,465

#3 #1 AND #2 379
