# Supplementary material for: Comparative analysis of revision causes between robotic-assisted and conventional manual unicompartmental knee arthroplasty: a systematic review and meta-analysis
Source: Knee Surg Relat Res. 2026 Feb 26;38:10. doi: 10.1186/s43019-026-00311-x (PMC12937531; doi:10.1186/s43019-026-00311-x)
Supplement: Supplementary file 2 — Additional file2 (DOCX 149 KB) Results of methodological assessment. [file 43019_2026_311_MOESM2_ESM.docx]

**Supplementary table.** The methodological quality of cohort studies in accordance with the Newcastle-Ottawa Scale (NOS).

| study | Selection | | | | Comparability | Outcome | | | Total score |
| --- | --- | --- | --- | --- | --- | --- | --- | --- | --- |
|  | Representativeness of the exposed cohort | Selection of the non exposed cohort | Ascertainment of exposure | Demonstration that outcome of interest was not present at start of study | Comparability of cohorts on the basis of the design or analysis | Assessment of outcome | Was follow-up long enough for outcomes to occur? | Adequacy of follow up of cohorts? |  |
| Constant Foissey  2023 | 1 | 1 | 1 | 1 | 2 | 1 | 1 | 1 | 9 |
| D.C. Hansen et al  2014 | 1 | 1 | 1 | 1 | 2 | 0 | 1 | 1 | 8 |
| G.Guild et al  2025 | 1 | 1 | 1 | 1 | 2 | 0 | 1 | 1 | 8 |
| Guido Maritan  2023 | 1 | 1 | 1 | 1 | 2 | 1 | 1 | 1 | 9 |
| Jason Wong  2019 | 1 | 1 | 1 | 1 | 2 | 0 | 1 | 1 | 8 |
| L.Andriollo  2024 | 1 | 1 | 1 | 1 | 2 | 0 | 1 | 1 | 8 |
| R. Canetti  2018 | 1 | 1 | 1 | 1 | 2 | 0 | 1 | 1 | 8 |
| St Mart  2020 | 1 | 1 | 1 | 1 | 2 | 0 | 1 | 1 | 8 |
| V. Rossi et al  2025 | 1 | 1 | 1 | 1 | 2 | 0 | 1 | 1 | 8 |

**Supplementary table.** The methodological quality of case-control studies in accordance with the Newcastle-Ottawa Scale (NOS).

| study | Selection | | | | Comparability | Outcome | | | Total score |
| --- | --- | --- | --- | --- | --- | --- | --- | --- | --- |
|  | Is the case definition adequate? | Representativeness of the cases | Selection of Controls | Definition of Controls | Comparability of cases and controls on the basis of the design or analysis | Ascertainment of exposure | Same method of ascertainment for cases and controls | Non-Response Rate |  |
| Batailler  2018 | 1 | 1 | 1 | 1 | 2 | 0 | 1 | 0 | 7 |
| Guillaume  2020 | 1 | 1 | 1 | 1 | 2 | 0 | 1 | 0 | 7 |
| Lau et al  2024 | 1 | 1 | 0 | 1 | 2 | 0 | 1 | 0 | 6 |
| Yeung et al  2023 | 1 | 1 | 1 | 1 | 2 | 0 | 1 | 1 | 8 |


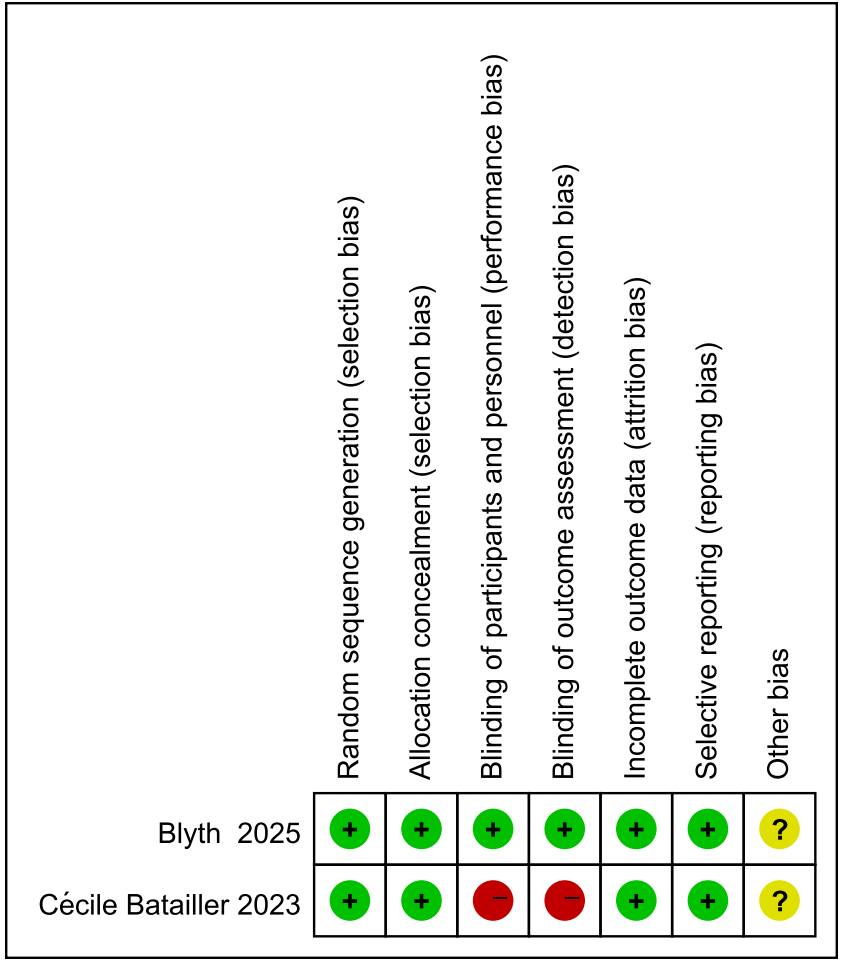


**Supplementary figure.** Risk of bias summary of randomized controlled trials.
