# Supplementary material for: Comparative analysis of revision causes between robotic-assisted and conventional manual unicompartmental knee arthroplasty: a systematic review and meta-analysis
Source: Knee Surg Relat Res. 2026 Feb 26;38:10. doi: 10.1186/s43019-026-00311-x (PMC12937531; doi:10.1186/s43019-026-00311-x)
Supplement: Supplementary file 3 — Additional file3 (DOCX 87 KB) Results of Begg’s test and Egger’s test. [file 43019_2026_311_MOESM3_ESM.docx]

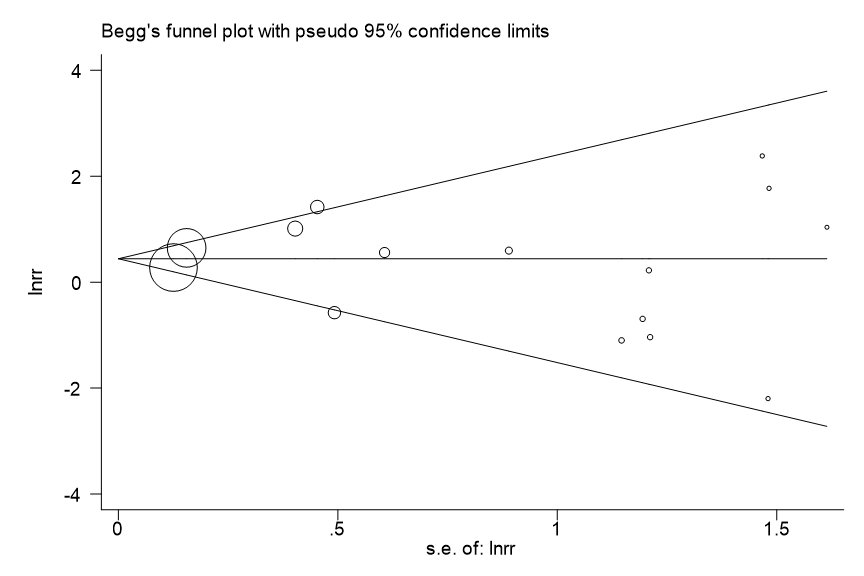


**Supplementary figure.** Begg’s test for total revision rate, with P value=0.767.


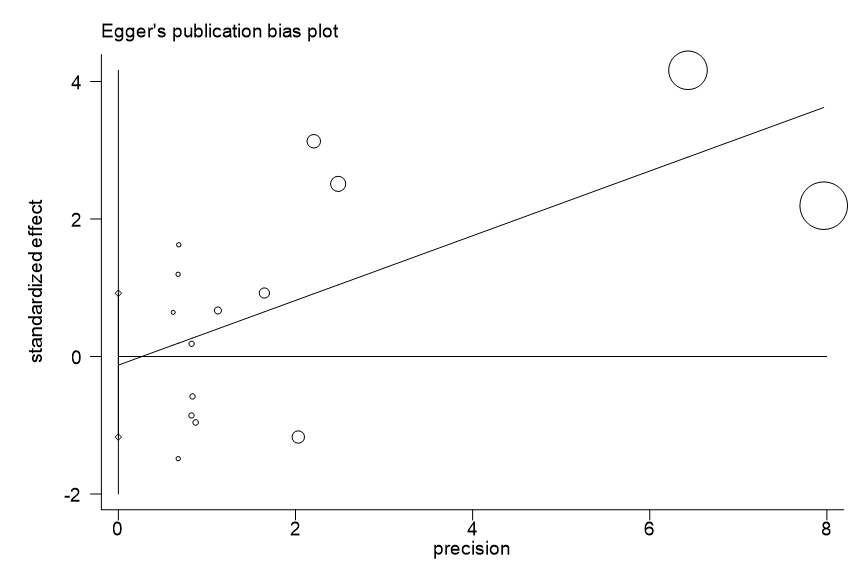


**Supplementary figure.** Egger’s test for total revision rate, with P value= 0.799.
