# Supplementary material for: Comparative analysis of revision causes between robotic-assisted and conventional manual unicompartmental knee arthroplasty: a systematic review and meta-analysis
Source: Knee Surg Relat Res. 2026 Feb 26;38:10. doi: 10.1186/s43019-026-00311-x (PMC12937531; doi:10.1186/s43019-026-00311-x)
Supplement: Supplementary file 4 — Additional file4 (DOCX 14 KB)Results of subgroup analysis [file 43019_2026_311_MOESM4_ESM.docx]

**Supplementary table.** Results of subgroup analysis of total revision rate according to different study type.

| Subgroup | Studies included | C-UKA | | R-UKA | | Risk ratio (95 % CI) | P value | Heterogeneity (P/I^2^) | Model |
| --- | --- | --- | --- | --- | --- | --- | --- | --- | --- |
|  |  | Events | Total | Events | Total |  |  |  |  |
| Case-control study | 4 | 30 | 427 | 17 | 412 | 1.77[1.00, 3.13] | 0.05 | 41% | Fixed effect |
| Cohort study | 9 | 642 | 21765 | 144 | 7300 | 1.57[1.31, 1.88] | <0.00001 | 44% | Fixed effect |
| RCT | 2 | 5 | 98 | 6 | 97 | 1.10[0.01, 97.97] | 0.03 | 79% | Random effect |
