# Supplementary material for: Comparative analysis of revision causes between robotic-assisted and conventional manual unicompartmental knee arthroplasty: a systematic review and meta-analysis
Source: Knee Surg Relat Res. 2026 Feb 26;38:10. doi: 10.1186/s43019-026-00311-x (PMC12937531; doi:10.1186/s43019-026-00311-x)
Supplement: Supplementary file 5 — Additional file5 (DOCX 21 KB) Ranking of the causes. [file 43019_2026_311_MOESM5_ESM.docx]

**Supplementary table.** Number of study reporting any specific reason for revision.

| Reasons | C-UKA | R-UKA |
| --- | --- | --- |
| Loosening | 10 | 8 |
| Progression of disease | 5 | 3 |
| Pain | 4 | 6 |
| Infection | 4 | 3 |
| Fracture | 2 | 2 |
| Malposition | 2 | 2 |
| Instability | 2 | 2 |
| Limb malalignment | 4 | 1 |
| Dislocation | 1 | 0 |
| Tear of meniscus | 1 | 0 |

**Supplementary table.** Ranking of the causes.

| Ranking |  | C-UKA |  |  |  | Ranking |  | R-UKA |  |  |
| --- | --- | --- | --- | --- | --- | --- | --- | --- | --- | --- |
|  | Reason | Number of revision, n | The proportion in all reasons (%) | Incidence within the first five years (%) |  |  | Reason | Number of revision, n | The proportion in all reasons (%) | Incidence within the first five years (%) |
| 1 | Loosening | 149 | 22.0 | 0.6 |  | 1 | Loosening | 30 | 18.2 | 0.3 |
| 2 | Progression of disease | 70 | 10.3 | 0.3 |  | 2 | Infection | 24 | 14.5 | 0.3 |
| 3 | Infection | 52 | 7.7 | 0.2 |  | 3 | Pain | 11 | 6.7 | 0.1 |
| 4 | Pain | 25 | 3.7 | 0.1 |  | 3 | Progression of disease | 10 | 6.1 | 0.1 |
| 5 | Limb malalignment | 22 | 3.4 | 0.1 |  | 5 | Instability | 4 | 2.4 | NA |
| 6 | Instability | 9 | 1.3 | NA |  |  |  |  |  |  |

NA: Not applicable.
