# Supplementary material for: Comparative analysis of revision causes between robotic-assisted and conventional manual unicompartmental knee arthroplasty: a systematic review and meta-analysis
Source: Knee Surg Relat Res. 2026 Feb 26;38:10. doi: 10.1186/s43019-026-00311-x (PMC12937531; doi:10.1186/s43019-026-00311-x)
Supplement: Supplementary file 6 — Additional file6 (DOCX 13 KB) Revision rates of UKA with different types of bearing. [file 43019_2026_311_MOESM6_ESM.docx]

**Supplementary figure.** Forest plot of total revision of the 4 studies exclusively evaluating fixed-bearing UKA.





**Supplementary figure.** Forest plot of total revision of the 11 studies mixing fixed-bearing UKA and mobile-bearing UKA together.
